# Venipuncture-Induced Complex Regional Pain Syndrome: A Case Report and Review of the Literature

**DOI:** 10.1155/2014/613921

**Published:** 2014-08-19

**Authors:** Foad Elahi, Chandan G. Reddy

**Affiliations:** ^1^Center of Pain Medicine, University of Iowa, 200 Hawkins Drive 5JPP, Iowa City, IA 52242, USA; ^2^Department of Neurosurgery, University of Iowa, Iowa City, IA, USA

## Abstract

Venipuncture, the most frequently performed invasive medical procedure, is usually benign. Generally it produces only transitory mild discomfort. Venipuncture-induced neuropathic pain is hard to recognize at an early stage. Medical literature reviews show that there is not adequate medical knowledge about this important subject. The inciting incident in complex regional pain syndrome (CRPS) can often seem far too trivial to result in a condition with such severe pathophysiologic effects. The practicing physician has little information available to enable early recognition of the condition, initiation of multidisciplinary treatment modalities, and proper referral to pain specialists. We encountered a unique case of venipuncture-induced complex regional pain syndrome (CRPS). The patient is a 52-year-old school teacher with no significant past medical history, who presented initially to the Center of Pain Medicine with left upper extremity pain. The pain started while phlebotomy was performed in the patient's left antecubital area for routine blood check. The patient's pain did not improve with multiple medications, physical therapy, or several nerve blocks. The patient demonstrated all the signs and symptoms of chronic neuropathic pain of CRPS in the upper extremity with minimal response to the continuous pain management. We decided to proceed with cervical spinal cord nerve stimulation along with continuing other modalities. The patient responded to this combination. During the follow-up, we noticed that the patient's pain course was complicated by extension of the CRPS to her lower extremity. We will describe the course of treatment for the patient in this paper. In this paper we will discuss the electrical neuromodulation as an important modality in addition to the multidisciplinary pain management for a patient with venipuncture-induced chronic neuropathic pain.

## 1. Introduction

Venipuncture, the most frequently performed invasive medical procedure, is usually benign, producing only transitory mild discomfort. Several case series and individual patient reports document nerve injuries consequent to the procedure. While such injuries are reportedly rare (1 : 21,000–1 : 26,700) some can be severe with permanent residua, the most disturbing being chronic neuropathic pain, complex regional pain syndrome type 2 (CRPS-II), or causalgia. Venipuncture-induced CRPS-II/causalgia is, perhaps, the most paradigmatic human neuropathic pain that can follow acute nerve trauma [1].

Complex regional pain syndrome (CRPS) is an enigmatic clinical condition characterized by various combinations of sensory, autonomic, and motor dysfunction. CRPS is diagnostically distinguished into two subtypes, CRPS-I (regional sympathetic dystrophy) which is triggered by a noxious event without known peripheral nerve injury, while CRPS-II (causalgia) is the result of known peripheral nerve injury.

The pain is continuous, not associated with a single nerve/dermatome, and is disproportionate in chronicity and/or severity to any potential inciting event. The syndrome shows variable progression over time. The pain is regional (not in a specific nerve territory or dermatome) and usually has a distal predominance of abnormal sensory, motor, sudomotor, vasomotor, and/or trophic findings. The resultant debilitating chronic pain affects the extremities and is associated with high rates of relapse [2].

The underlying pathophysiology remains controversial and poorly understood, with no evidence that the two subtypes differ in causative mechanisms. Over the past few years there have been increasing evidence and acceptance of a multifactorial etiology. The mechanisms include both peripheral and central sensitization, inflammation, sympathetic dysregulation, somatosensory cortex reorganization, genetic factors, and psychophysiologic interactions. Adding to the underlying complexity in diagnosing and treating CRPS, each patient's symptoms are likely a result of different combinations of mechanism that can change over time [3].

Original International Association for the Study of Pain (Orlando) diagnostic criteria for complex regional pain syndrome include (1) the presence of an initiating noxious event or a cause of immobilization, (2) continuing pain, allodynia, or hyperalgesia with which the pain is disproportionate to any inciting event, (3) evidence of edema, changes in skin blood flow, or abnormal sudomotor activity in the region of pain at some point in time, and (4) diagnosis that is excluded by the existence of conditions that would otherwise account for the degree of pain and dysfunction.

The inciting incident in CRPS can often seem far too trivial to result in a condition with such severe pathophysiologic effects. Needlestick causing distal nerve injury has been shown to produce CRPS-like symptoms in rat models, which suggests that similar pathophysiologic mechanisms may contribute to the development of CRPS after venipuncture in humans [4]. In a Japanese study, between 2004 and 2008, venipunctures were performed with 133 cases of resultant persistent pain and 19 cases of neuropathic pain [5].

## 2. Case Presentation

A 52-year-old right handed female presented with a chief complaint of left arm and left hand pain. The patient's symptoms began after a phlebotomist had a difficult time obtaining blood draws from the patient's left antecubital region for a cholesterol test. The patient described that immediately following the needle insertion it felt like the patient was being “electrocuted.” A few days later the patient went to the doctor due to this ongoing pain. The patient developed a shooting, burning, and constant neuropathic pain. The pain was originally in the left thumb finger and gradually extended to the indicis and middle finger with the relatively rapid expansion of the entire hand up to the wrist and sharp radiating pain distally from the elbow. The pain was somewhat diffused and was most sensitive along all the dermatomes below the elbow. Due to the continuation of her pain and with no benefit from initial pain medications regimen, her pain specialists decided to do stellate ganglion block, epidural steroid injections, along with physical therapy, occupational therapy, and multiple combined pain medications. She never experienced a tolerable pain level during the use of her medications even in the highest permitted dose. The patient had tried gabapentin, tramadol, baclofen, amitriptyline, hydrocodone, nucynta, savella, and lyrica, as well as lidoderm patches for her symptoms.

The patient presented to the pain clinic with pain and rated the pain as 8 out of 10 on visual analog scale and stated that the pain was perceived constantly around moderate to severe. The patient reported a constant warm and burning sensation. The patient had difficulty falling asleep, as well as interrupted sleep due to change of position and inadvertent pressure on the left arm. The patient is right handed but due to pain was able to use the left arm for daily activities.

The patient did not complain of any associated neurological symptoms. However, the patient stated that she has become weaker in her left upper extremity secondary to the lack of use from the pain. The patient's other symptoms are associated with redness, bluish discoloration, temperature disparity amongst contralateral upper extremities, and allodynia.

After one year since the inciting event and trial of multimodality treatment we decided to try dorsal spinal neuromodulation. During seven days of a spinal cord stimulation trial, the patient was able to perform many daily activities with reasonable pain control. The patient reported the pain between 2 and 3 on the visual analog scale during the trial period. The patient was deemed to be a candidate for permanent implantation of dorsal column stimulator. The patient underwent the implantation of 8-contact-compact-lead percutaneously sensor rechargeable battery (2 × 8 octad leads, model #3778-75 Medtronic Co.).

**Figure 1:**
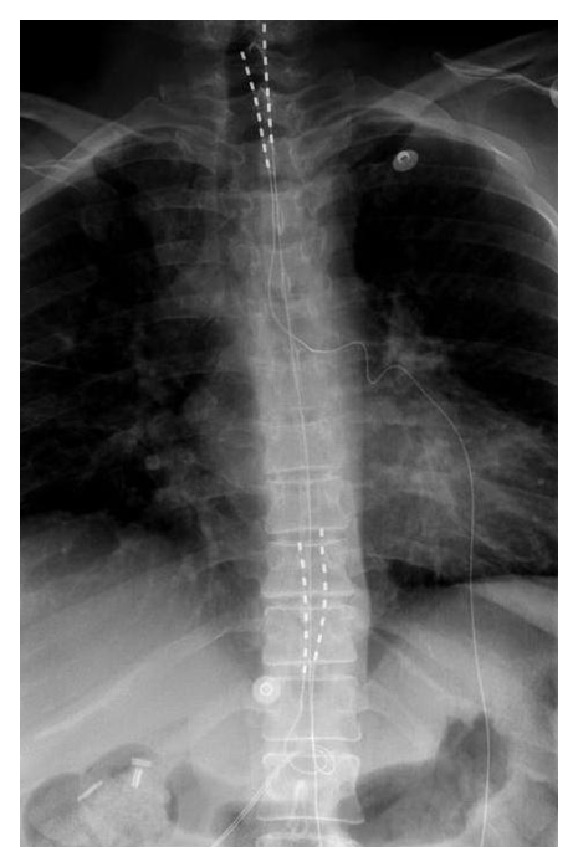
In this X-ray picture you will see the cervical and thoracic leads.

On a six-month follow-up, the patient reported left foot pain and discoloration. The patient's CRPS symptoms had now spread to involve the right lower extremity. We administered an aggressive course of physical therapy with desensitization techniques along with occasional lumbar sympathetic blocks and pain behavioral modifications techniques with multimodal medication management. The symptoms remained unresponsive and had been refractory to the multimodality management. The patient reported constant pain in the right leg with severity of 8 on visual analog scale. Gradually skin discoloration and nail changes along with hair loss on the right foot created a clear picture of CRPS. Interestingly, the patient reported great pain relief on the upper extremity by using a cervical spine stimulator. The patient was able to use the upper extremity for daily activities. The patient's right leg pain was greatly disabling and due to the unresponsive CRPS symptoms we decided to proceed with thoracic dorsal column stimulator placement after the patient demonstrated a great response to 7 days of the trial. A thoracic spinal cord stimulation implant was done via percutaneous placement of 2 × 8 octad leads (Medtronic Co.) (see (Figure 1)).

We did six-month follow-up after the patient's last surgery. The patient was 100% satisfied with the pain management result. We interrogated the spinal cord device. The patient was able to manage to go back to her school job using neuromodulation 100% of the time along with moderate consumption of gabapentin and tramadol. The patient rated her pain severity at both upper and lower extremities at a 2 on visual analog scale.

## 3. Discussion

In this paper, we presented a severe and extreme case with venipuncture-induced CRPS. Pain is the chief reason why patients attend to doctors. The recognition of neuropathic pain after inadvertent injury to a nerve during operation should be straightforward. It is associated with disturbance of sensation and power and is expressed in the distribution of the nerve, remote from the site of operation. The recognition of neuropathic pain after a venipuncture is more difficult. Venipuncture, the most frequently performed invasive medical procedure, is usually benign. Generally it produces only transitory mild discomfort. Venipuncture-induced neuropathic pain is hard to recognize at early stage.

The injury may induce numbness or diffuse pins and needles through the injured limb for the first hour or two, or the pain from the multiple trials for venous access may mask the underlying nerve pain. On the other hand, neuropathic pain is sometimes so severe that the patient is scarcely aware of the injury. Spontaneous sensory symptoms which are usually painful, perceived in the limb, the hand, or the foot are significant and should not be overlooked.

We have been interested to hear patients describe their pain following accidental damage to the nerve. The exact timing of injury is well known to the patient.

We used the patient's description along with visual analog scale to document the patients' pain score during the follow-up. There is a vogue for measuring pain by visual analogue scales (VAS), a system which too often gives a spurious sense of objectivity. However, Kato et al. (2006) found a strong concordance between the VAS linear scale and the peripheral nerve injury scale [6].

Therapies included analgesic, anti-inflammatory, tricyclic antidepressant, anticonvulsant medications, transcutaneous electrical nerve stimulation, nerve blocks, stellate ganglion blockades, and using chemical, thermal, or surgical sympathectomy.

CRPS usually remains restricted to one limb, but it can spread to other body parts. CRPS in one limb may extend to another limb either as a result of a new trauma to a previously unaffected limb or because the syndrome spreads spontaneously. The severity of CRPS symptoms in the second limb may not differ significantly from that in the first limb. The mechanism underlying spontaneous spread of CRPS to other limbs is unclear. Venipuncture-induced CRPS patients raise many unanswerable questions regarding incidence, predisposition, and causation of disabling nerve injuries.
